# High health literacy is associated with less obesity and lower Framingham risk score: Sub-study of the VGH-HEALTHCARE trial

**DOI:** 10.1371/journal.pone.0194813

**Published:** 2018-03-28

**Authors:** Yuan-Lung Cheng, Jiah-Hwang Shu, Hsiu-Chuan Hsu, Ying Liang, Ruey-Hsing Chou, Pai-Feng Hsu, Yuan-Jen Wang, Yaw-Zon Ding, Teh-Ling Liou, Ying-Wen Wang, Shao-Sung Huang, Chung-Chi Lin, Tse-Min Lu, Hsin-Bang Leu, Shing-Jong Lin, Wan-Leong Chan

**Affiliations:** 1 School of Medicine, National Yang-Ming University, Taipei, Taiwan; 2 Taipei Municipal Gan-Dau Hospital, Taipei, Taiwan; 3 Department of Nursing, Taipei Veterans General Hospital, Taipei, Taiwan; 4 Healthcare and Management Center, Taipei Veterans General Hospital, Taipei, Taiwan; 5 Cardiovascular research center, National Yang-Ming University, Taipei, Taiwan; 6 Institute of public health, National Yang-Ming University, Taipei, Taiwan; University of Oslo, NORWAY

## Abstract

**Backgrounds:**

Lower health literacy (HL) is associated with several cardiovascular disease (CVD) risk factors such as diabetes, hypertension, and metabolic syndrome (MS). The aim of our study was to investigate the association between HL and the Framingham 10-year risk score of CVD.

**Methods:**

From 2015–2016, 1010 subjects aged 23 to 88 years receiving health check-up in Taipei Veterans General Hospital had complete clinical evaluations and laboratory examinations. Fatty liver was diagnosed by ultrasonography. The short form questionnaire adapted from the Mandarin Health Literacy Scale was used to assess HL. The Framingham risk score was calculated by patient characteristics.

**Results:**

Subjects with higher BMIs were associated with lower HL scores. The proportion of subjects with MS was higher in the lower health literacy score group (≤ 9) at 28.8%; further analysis found that lower HL was significantly associated with MS in women but not in men. The Spearman’s *rho* demonstrated that the HL score was significantly associated with the BMI-based (*rho* = -0.11; *P* < 0.001) or lipid-based (*rho* = -0.09; *P* < 0.004) Framingham risk score.

**Conclusions:**

Higher HL scores were associated with less CVD risk such as lower BMIs, less MS in women, and less fatty liver disease. Furthermore, HL had an inverse association with the Framingham risk score as expected. Therefore, HL in patients with CVD risk should be improved and considered as an important issue in terms of CVD reduction.

## Introduction

Ischemic heart disease and cerebrovascular disease are associated with an increased risk of morbidity and mortality and are the leading cause of death worldwide [1]. These diseases are caused by inappropriate lifestyle factors, and aggressive intervention therapies and consultation targeting risk factors such as obesity, cigarette smoking, physical inactivity, hypertension, hyperlipidemia, diabetes, and etc. have improved the outcome of CVD [2]. Recent systematic analysis for the Global Burden of Disease Study revealed age-standardized death rates of ischemic heart disease and cerebrovascular disease decreased 11.6% and 21.0% [1]. This showed an improvement in risk reduction, but there is still a great gap in achieving an acceptable result.

Health literacy (HL) is the ability to communicate and understand basic health information to make appropriate health decisions concerning health care and disease prevention [3]. Although it is essential for good health, subjects with inadequate HL are common. Indeed, more than 33% of Americans had limited HL according to a national survey of HL in the United States, and 35% of respondents across the eight countries in the European Health Literacy Survey had problematic HL while about 30% of adults in Taiwan had low HL [3–5]. Challenges include a lack of familiarity with medical terms, difficulties in interpreting test values, and various educational backgrounds. These factors limit improved HL in the general population. To promote HL, the WHO recommends establishing policies to enforce plain-language approaches to health, and reframing limited HL as a challenge to healthcare systems. Health care providers should be trained to communicate more efficiently with people with low HL to enable them to access health services, understand health-related information, and make informed decisions [3].

The association between HL and risk factors for CVD such as hypertension and diabetes was studied previously with inconsistent results. Most studies showed that adequate HL was related with less hypertension occurrence [6–9], and patients with adequate HL had better blood pressure (BP) control [10–13] suggesting a close relationship between HL and hypertension. However, other studies showed that BP control was unrelated to HL status [14] and might be associated with a higher BP among subjects with adequate HL [15]. Studies of diabetes prevalence suggested that lower HL was independently associated with higher prevalence of diabetes [14, 16, 17]. Others concluded that glycemic control was positively correlated with HL status [18–23]; alternative studies found no such relationship [24–26].

Though evidence about the association between HL and HTN/diabetes is inconsistent, Health Literacy Solid Facts published by the World Health Organization (WHO) suggests that HL is an important factor in preventing non-communicable diseases such as heart disease and diabetes. These are associated with multiple modifiable risk factors [3]. To the best of our knowledge, only one study has analyzed the association between HL and the Framingham 10-year risk score of CVD, and it showed that HL was associated with a lower risk of CVD only in women. Furthermore, the study population was more than 80% Caucasians [27]. Therefore, we conducted this study to investigate whether HL was associated with the Framingham risk score and other related CVD risk factors in an Asian population.

## Materials and methods

### Study population

From February 2015 to January 2016, 7762 subjects received health check-up service provided by the internists in the Healthcare and Management Center, Taipei Veterans General Hospital. After explanation, 1100 questionnaires were sent to those who were willing to participate in the study, and all of them filled out the questionnaires in an hour at noon without assistance. All of them received complete clinical evaluations and laboratory examinations. Body mass index (BMI) was calculated as the weight in kilograms divided by the square of the height in meters. BP was measured after subjects had been seated for more than 5 minutes; the means of three consecutive readings were recorded as systolic and diastolic BP with a difference in systolic BP < 10 mmHg. Three of the following five abnormal findings were required for a diagnosis of MS according to the joint interim statement of the International Diabetes Federation Task Force on Epidemiology and Prevention [28]: high waist circumference (WC, men ≥ 90 cm or women ≥ 80 cm), triglyceride levels ≥ 150 mg/dL, low high-density lipoprotein-cholesterol (HDL) levels (men < 40 mg/dL or women < 50 mg/dL), systolic BP ≥ 130 mmHg and/or diastolic BP ≥ 85 mmHg, and fasting glucose ≥ 100 mg/dL. Ultrasonography was performed with a Prosound Alpha 7 device (Aloka, Prosound Alpha 7, Tokyo, Japan) to diagnose fatty liver disease in 945 subjects; the diagnosis of fatty liver disease was based on the practice guideline of the American Gastroenterological Association [29]. This study followed the standards of the Declaration of Helsinki and was approved by the Institutional Review Board of Taipei Veterans General Hospital. Written informed consent was obtained from all study subjects.

### Health literacy

A validated 11-item short form adapted from the Mandarin Health Literacy Scale (s-MHLS) focusing on reading comprehension and numeracy was used to assess individuals’ HL in terms of their ability to read and understand health information. The 11 items were extracted from two sections of the original MHLS: outpatient dialogues (four items) and prescription labels (seven items). Of those 11 items, three assessed numeracy skills and eight assessed reading comprehension. All of them were three-option multiple choice questions with only one correct option for each question. If no option or more than one option was written, the answer was graded as wrong. The score was the sum of the correct answers. (Range: 0–11) The Cronbach’s correlation between the MHLS and s-MHLS was 0.97, and the s-MHLS had satisfactory internal reliability of 0.94 and a mean score of 9.01 in the validation population. [30, 31]

### The Framingham risk score

The score was calculated as a function including age, SBP, antihypertensive use, current smoking, diabetes, and total cholesterol and HDL (for cholesterol-based risk) or BMI (for BMI based risk). The score estimates 10-year absolute CVD risk including coronary death, myocardial infarction, coronary insufficiency, hemorrhagic stroke, and heart failure. [32]

### Biochemical and serological markers

Venous blood samples were collected after an overnight fast. Serum biochemical markers were measured with a TBA-c16000 automatic analyzer (Toshiba Medical Systems, Tochigi, Japan). Glycated hemoglobin (HbA1c) was measured with a G7 or G8 high-performance liquid chromatography analyzer (Tosoh Bioscience, Inc., South San Francisco, California).

### Statistical analysis

The demographics of the study subjects were summarized as numbers with percentages for categorical variables and as means with standard deviations for continuous variables after the subjects were divided into three groups based on HL scores. Comparisons among the three groups were performed by Pearson’s chi-squared test and one-way analysis of variance (ANOVA) for categorical and continuous variables, respectively. If *P* value was significant (*P* < 0.05) in ANOVA, then a post hoc Scheffe test was performed to make pairwise comparisons. The Bonferroni correction for multiple comparisons was used as the post hoc test following Pearson’s chi-squared test to reduce the chance of type I errors; the corrected critical *P* value was 0.017. The Spearman’s rank correlation coefficients were calculated to indicate the direction of the relationships between the variables. The Bonferroni correction for multiple comparisons was performed to reduce the chance of type I errors; the corrected critical *P* value was 0.017. Statistical analysis used STATA version 13.0 (Stata Corp., College Station, TX, USA).

## Results

### Subject characteristics

The mean age of all subjects was 51.0 years ranging from 23 to 88; 62.0% of these were men. The demographic data of all subjects was summarized in Table 1 divided by three groups based on HL scores. The mean ages of subjects with HL ≤ 9, HL = 10, and HL = 11 were 55.7, 50.5, and 50.4 years, respectively. There were more males in the group with HL scores lower than 9 points, and this group was also older. They had higher BMIs, systolic BP, HbA1c, fasting glucose, ALP, GGT, WC, and a lower rate of drinking.

**Table 1:** Subject demographics based on health literacy score.

	HL<= 9 (111)	HL = 10 (267)	HL = 11 (632)	*P* value
Age, years*	55.7±11.1^&^^	50.5±12^&^	50.4±10.9^	<0.001
**Gender (M/F) (**%**)**	82/29^&^^ (73.9/26.1)	163/104^&^ (61.1/38.9)	381/251^ (60.3/39.7)	0.023
**HTN history (yes/no) (**%**)**	28/83 ^&^ (25.2/74.8)	35/230 ^&^ (13.2/86.8)	120/505 (19.2/80.8)	0.014
Height, cm*	166.3±8.2	166.6±8.7	166.4±7.9	0.928
Weight, kg*	70.2±12.5^&^^	66.3±12.5^&^	66.3±12.1^	0.008
**BMI, kg/m**^2^*	25.3±3.8^&^^	23.8±3.3^&^	23.9±3.4^	<0.001
WC, cm*	89.9±12.3 ^&^^	87.1±13.9^&^	86.6±12.5^	0.047
SBP, mmHg*	125.3±17.7^&^^	119.9±17.2^&^	120.1±18.1^	0.014
DBP, mmHg*	76.6±10.4	75.2±11.2	76.3±11.5	0.319
**Smoking (yes/no) (**%**)**	30/80 (27.3/72.7)	60/207 (22.5/77.5)	130/502 (20.6/79.4)	0.278
**Drinking (yes/no) (**%**)**	48/62 ^ (43.6/56.4)	83/184 (31.1/68.9)	198/434 ^ (31.3/68.7)	0.033
Cholesterol, mg/dL*	196.7±38.7	202.6±35.9	202.9±35.9	0.238
Triglyceride, mg/dL*	118.1±58.9	117.1±63.6	122.6±75.3	0.522
Uric acid, mg/dL*	6.5±1.7	6.4±1.6	6.3±1.6	0.26
HDL, mg/dL*	47.8±12.5	49.7±13.8	49.3±12.9	0.445
LDL, mg/dL*	120.8±32.7	126.5±34.1	126.3±32.4	0.24
Cholesterol/HDL*	4.3±1.1	4.3±1.3	4.3±1.2	0.88
LDL/HDL*	2.7±0.8	2.7±1	2.7±1	0.678
Non-HDL cholesterol, mg/dL*	148.8±37	152.9±36.1	153.6±35.5	0.433
**HbA1c (**%)*	5.9±0.9^&^^	5.6±0.7^&^	5.7±0.7^	0.001
Fasting glucose, mg/dL*	102.8±32.4^&^^	90.8±16.9^&^	93.4±21.2^	<0.001
AST, IU/L*	26.2±14.4	24±9	23.7±10.9	0.089
ALT, IU/L*	31.3±23.9	27.2±18.7	27±20.4	0.119
ALP, IU/L*	68.1±19.3^&^^	62.6±15.9^&^	62.3±16.3^	0.003
GGT, IU/L*	31.1±27.9^&^^	24.8±14.7^&^	25.5±17.5^	0.005
TB, mg/dL*	1.1±0.5	1.2±0.6	1.1±0.5	0.271
Creatinine, mg/dL*	0.9±0.2	0.9±0.2	0.9±0.2	0.394

HL, health literacy; M, male; F, female; HTN, hypertension; BMI, body mass index; WC, waist circumference; SBP, systolic blood pressure; DBP, diastolic blood pressure; HDL, high-density lipoprotein; LDL, low-density lipoprotein; HbA1c, glycated hemoglobin; AST, aspartate aminotransferase; ALT, alanine aminotransferase; ALP, alkaline phosphatase; GGT, gamma-glutamyltransferase; TB, total bilirubin.

*Expressed as mean ± standard deviation.

^&^Indicating significant post hoc analysis between HL<= 9 and HL = 10.

^Indicating significant post hoc analysis between HL<= 9 and HL = 11.

^%^Indicating significant post hoc analysis between HL = 10 and HL = 11.

### The association between HL and BMI/fatty liver disease

**Fig 1:**
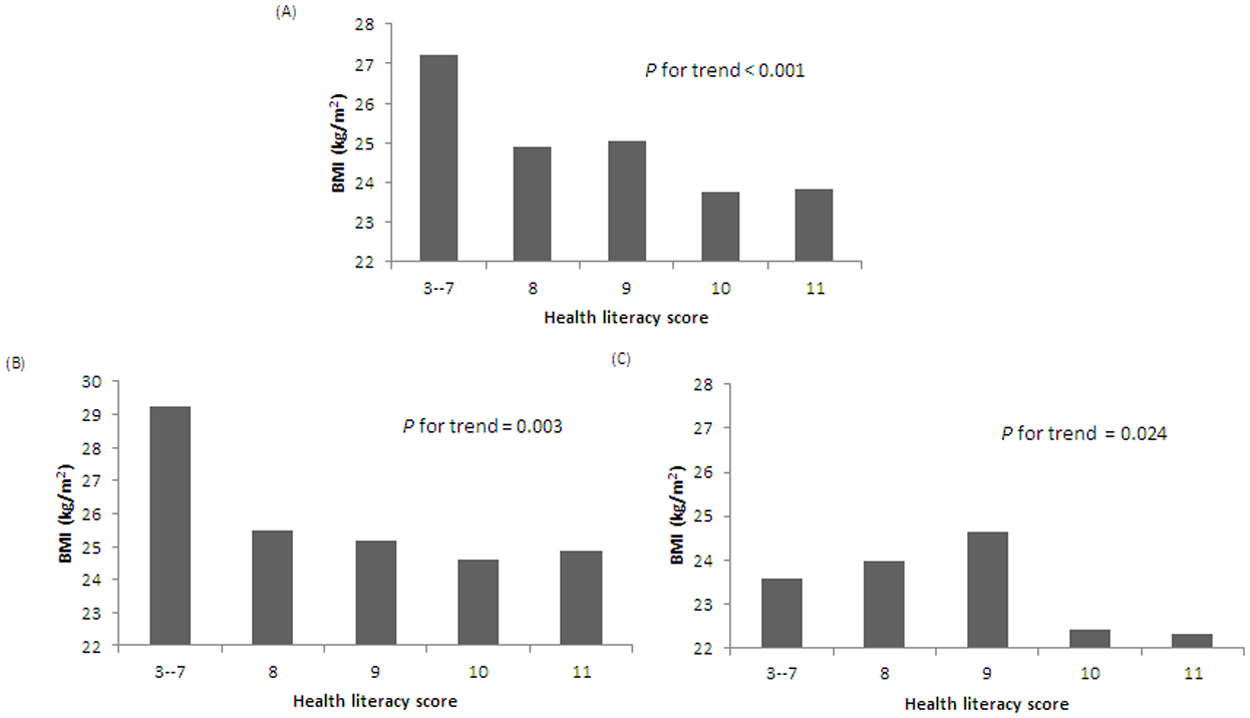
The association between health literacy score and BMI: (A) all subjects, (B) men, (C) women.

The BMI of subjects averaged 23.99, and the HL≤ 9 group was significantly associated with a higher BMI than the other two groups (25.3 vs 23.8 and 23.9 kg/m^2^ in the HL = 10 group and the HL = 11 group, respectively). Subjects with higher BMIs were associated with lower HL scores (*P* for trend < 0.001). This trend existed for both men and women (*P* for trend = 0.003 and 0.024, respectively) (Table 1 and Fig 1).

**Fig 2:**
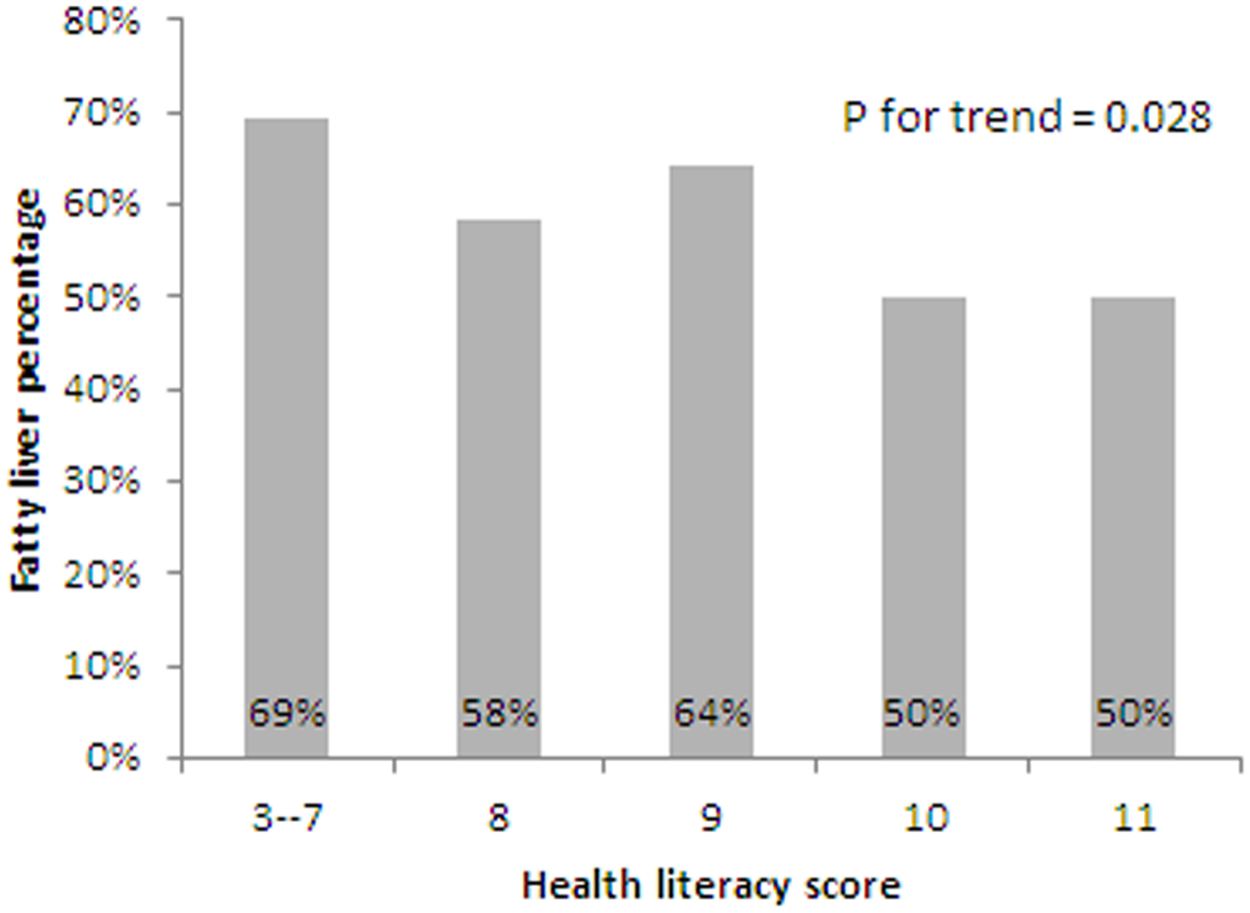
The association between health literacy score and fatty liver disease.

Of the 945 subjects receiving abdominal ultrasonography, 51% were diagnosed with fatty liver disease. The HL≤ 9 group had a significantly higher rate of fatty liver disease than the other two groups (63.6% vs 49.8 and 49.9 in the HL = 10 and HL = 11 groups, respectively). The trend showed an inverse association between HL scores and fatty liver disease; this was significant as well (*P* = 0.028) (Table 2 and Fig 2).

**Table 2:** The prevalence of metabolic syndrome and fatty liver disease in different HL groups.

	HL<= 9 (111)	HL = 10 (267)	HL = 11 (632)	*P* value
**Metabolic syndrome (yes/no) (**%**)**	32/79 (28.8/71.2)	61/203 (23.1/76.9)	143/481 (22.9/77.1)	0.392
**Fatty liver disease (yes/no) (**%**)**	68/39^&^^ (63.6/36.5)	123/124^&^ (49.8/20.2)	295/296^ (49.9/50.1)	0.029

HL, health literacy.

^&^Indicating significant post hoc analysis between HL<= 9 and HL = 10.

^Indicating significant post hoc analysis between HL<= 9 and HL = 11.

^%^Indicating significant post hoc analysis between HL = 10 and HL = 11.

### The association between HL and MS

**Fig 3:**
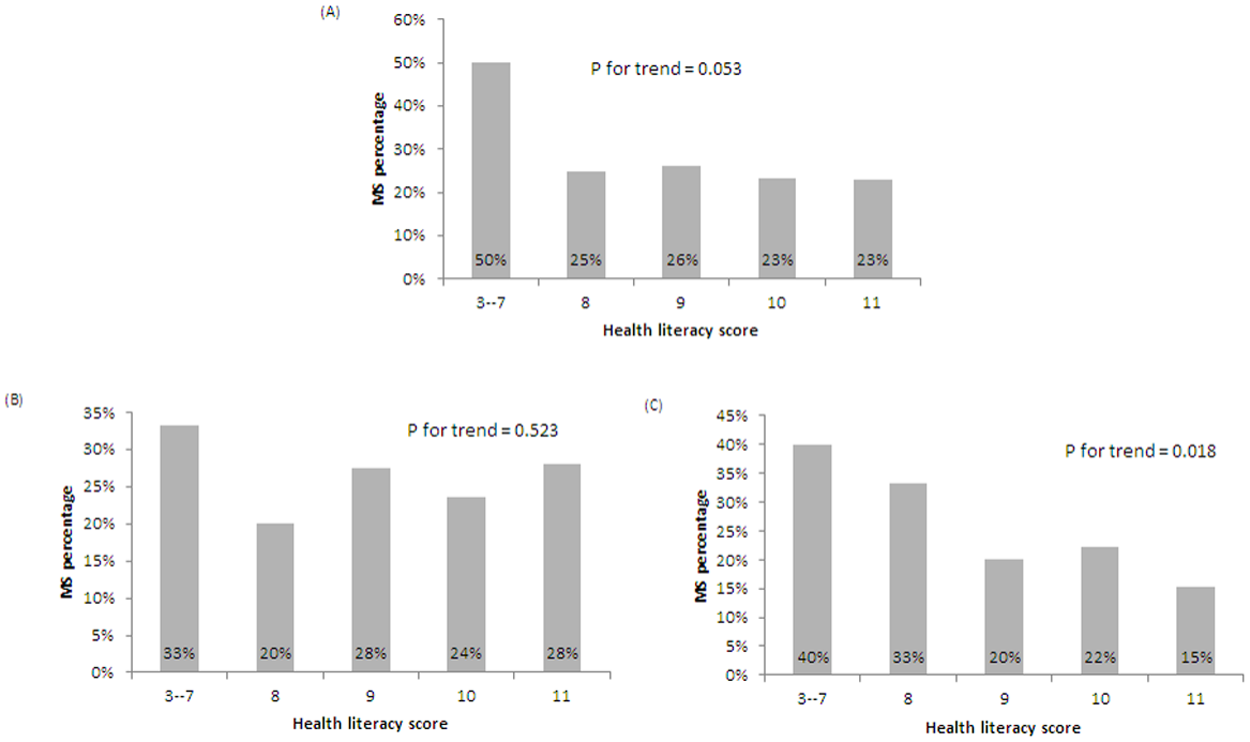
The association between health literacy score and metabolic syndrome: (A) all subjects, (B) men, and (C) women.

MS was diagnosed in 23.6% of subjects: 18.1% of women and 27.1% of men. The data for WC was missing in 56 subjects (the diagnosis of MS could not be determined in 11 subjects and they were excluded in this analysis). The proportion of subjects with MS was higher in the HL ≤ 9 group (28.8%). The *P* for trend between MS and HL was marginally significant at 0.053. Further analysis found female subjects with a lower HL was significantly associated with more MS; the trend between HL and MS was insignificant in men (Fig 3).

### The association between Framingham 10-year CVD risk and HL score

The BMI-based Framingham 10-year CVD risk averaged 11.85% while the lipid-based Framingham 10-year CVD risk score averaged 11.46%. A significant negative correlation was found between HL and the BMI-based Framingham 10-year risk (*rho* = -0.11; *P* < 0.001). The inverse association was also noted between HL and the lipid-based Framingham CVD risk (*rho* = -0.09; *P* < 0.004). (Table 3)

**Table 3:** Association among cardiovascular disease risk and health literacy score.

**Spearman’s *rho***	**Framingham score** **BMI-based**	**Framingham score** **lipid-based**	**HL**
**Framingham score** **BMI-based**	1		
**Framingham score** **lipid-based**	0.9773*	1	
**HL**	-0.1096*	-0.0946*	1

HL, health literacy.

*P < 0.017 (Bonferroni correction).

## Discussion

Our study showed that HL was inversely associated with CVD risk factors including BMI, fatty liver disease, and MS in women; HL also had a negative association with the Framingham risk score. Therefore, it is important to improve HL in general patients to reduce future risk of developing cardiovascular disease.

HL is a person’s ability to read, understand and act on medical instructions. Different scales focusing on variable aspects were used for HL evaluation [33]. The Rapid Estimate of Adult Literacy in Medicine (REALM) [6, 11, 16, 21, 22] focuses on health word recognition and pronunciation, the Test of Functional Health Literacy in Adults (TOFHLA) [10, 23, 25] and the Korean Functional Health Literacy test (KHLT) [7] test numeracy and reading comprehension, the Single Item Literacy Screener (SILS) [14] and the Brief Health Literacy Screen (BHLS) [9, 12, 15] are self-reported survey tests to identify HL status, the New Vital Sign (NVS) [34] evaluates the ability to read a nutrition label, and the HBP health literacy scale [35] and the Diabetes Numeracy Test [21] are disease-specific HL scales.

More recently assessment tools including the European Health Literacy Survey Questionnaire (HLS-EU-Q) [17] and the nine-domain Health Literacy Questionnaire (HLQ) have been developed to cover more aspects of HL [36]. Because various scales were used, the associations of HL to diseases such as hypertension and diabetes are inconsistent. A cross-sectional study revealed that patients with adequate HL had a systolic BP that was 6.1 mmHg lower than patients with limited HL [6]. Two more studies—one of which was conducted in Korean elderly patients using the KHLT and another in middle-aged Japanese adults—demonstrated that lower HL was associated with higher BP [7, 8]. Other studies have reported that higher HL is associated with better BP control [10, 37] suggesting the close association between low HD and hypertension.

However, Tiller et al. reported no such association between hypertension and HL as assessed by the HLS-EU-Q16 [17]. Willens et al. also found that higher health literacy assessed by the BHLS was associated with a small increase in BP.[15] A similar inconsistent association was also shown in diabetes patients. McNaughton et al. found that low HL as measured with the SILS was associated with higher HbA1c and random blood glucose [14]. Sudore et al. reported that limited HL (assessed by the REALM) was independently associated with higher prevalence of diabetes [16], and Tiller et al. showed that adequate HL was associated with less diabetes [17]. However, insignificant association between low HL and diabetes were also reported in other studies [24–26].

Various HL assessment tools, education backgrounds, and different abilities to understand health-related information, acquire medical counseling supports, and adhere to medications might contribute to the complexity and inconsistent association to health literacy [38]. Here, we clearly demonstrated the significant association between HL and cardiovascular disease associated risk factor and future risk of developing adverse event using s-MHLS, which is a modified MHLS with good correlation and reliability (Cronbach’s correlation between the MHLS and s-MHLS was 0.97; the s-MHLS has satisfactory internal reliability of 0.94 and a mean score of 9.01 in the validation population) [30, 31].

The association of weight and HL was also explored in previous studies. Lam et al. evaluated HL of 1035 children between 12 and 16 years old by the Chinese version of the sTOFHLA. This study showed that low HL was associated with being overweight and obese in adolescents (*p* = 0.017) [39]. Lassetter et al. used the NVS to evaluate HL among 354 Native Hawaiians and Pacific Islanders aged 18 years or older and showed that the BMI and NVS scores had a significant negative correlation (r = -0.12, *p* = 0.027) [34], while another cohort study from Geboers et al. showed that the association existed in older adults as well (ORs > 1.31, p-values < .005) [40]. This study found that higher BMIs were associated with lower HL, and the trend was significant in both genders comparable to previous studies (Fig 1).

Two other studies concluded that individuals with higher HL had a better outcome in weight loss programs. The first studied a stepped approach to weight loss and showed that participants who needed to be stepped up for counselling and meal replacement after failing to achieve weight loss goals were lower in HL (*p* = 0.03) [41]. The other study focused on diet and physical activity and revealed that HL moderated weight loss effects (*p* = .014). Participants with higher HL significantly lost more weight (*p* < .047) [42]. Accordingly, the HL could be an important evaluation for keeping ideal body weight and should be considered in weight loss programs—individuals with lower HL require more intensive counseling and education.

A recent analysis of the US population from 2003 to 2012 showed that the prevalence of MS is nearly 35% in the United States [43]. While the prevalence of MS was approximately 12% between 1999 and 2002 according to a community study in north Taiwan, it was much higher in this study (23.6%) and close to our previous study collecting information from subjects between 2002 and 2009 at 28.8% [44]. Westernization of diets, greater awareness of the syndrome, and higher socioeconomic status of the subjects might underlie this trend [45]. Furthermore, our findings suggested a negative association of HL and MS in women; women tend to have a healthier lifestyle, and this tendency might be reinforced with better HL. However, Yokokawa et al. showed that HL was inversely associated with MS among men instead of women. The prevalence of MS in all participants, men, and women was 15.6, 20.9, and 11.6%, respectively [46]. Different cohorts and HL scales might explain this discrepancy.

Non-alcoholic fatty liver disease (NAFLD) had been regarded as an incidental pathologic finding in type 2 diabetes and obesity; however, it was strongly related to features of MS and is even considered to be included in the definition of MS [47]. Yki-Jarvinen et al. suggested that NAFLD could be a more direct predictor of type 2 diabetes, CVD, and non-alcoholic steatohepatitis than MS [48]. While our study only showed a negative association between MS and HL in the women, the results revealed higher HL was closely related with less fatty liver disease. However, the lack of detailed alcohol use prevented us from excluding alcoholic fatty liver disease. Further studies are needed to validate the correlation.

HL has been associated with several CVD risk factors, and Martin et al. assessed the association between HL and CVD risk using subtests of the Woodcock Johnson III Tests of Achievement. This showed that HL—especially numeracy and listening comprehension—was related to the CHD risk. However, the association was only significant for women [27]. The study by Martin et al. enrolled 409 participants in their mid-40s: 80% were Caucasians and only 40% were men. Our results showed that HL determined by the s-MHLS had a significant association with CHD risk. Our cohort was aged 23 to 88, and 62% were men. Different tests and populations could be responsible for the slight discrepancy in the results.

There are several limitations in our study. First, most subjects had HL scores of 10 and 11, which might be because subjects receiving health check-up services tend to have higher education and socioeconomic status. Thus, the result could not be widely applied. Second, alcohol consumption was self-reported as yes, no, or having quit. In the ‘Yes’ group, the amount and the frequency of alcohol consumption was not documented, which prevented further analysis. Nevertheless, the prevalence of alcohol dependence is estimated to be only 3% in Taiwan, and thus likely has only a minor impact on the results [49]. Third, the causal relationship could not be determined due to the cross-sectional design—further longitudinal studies are required to further elucidate the issue.

In conclusion, HL was inversely associated with CVD risk factors namely BMI, fatty liver disease, and MS in women. Further analysis revealed that HL also had an inverse association with the Framingham risk score. Therefore, improving HL in patients with CVD risk is an important issue for CVD reduction.
